# Parent and child opinion on the use of standing desks in the classroom

**DOI:** 10.1016/j.pmedr.2024.102875

**Published:** 2024-08-30

**Authors:** Katie L. Wasserstein, Meghan L. Shah-Hartman, W. Gavin Luzier, Eric W. Schaefer, Mark E. Benden, Deepa L. Sekhar

**Affiliations:** a Department of Pediatrics, Penn State College of Medicine, Hershey, PA, United States; b Department of Public Health Sciences, Penn State College of Medicine, Hershey, PA, United States; c Department of Environmental and Occupational Health, Texas A&M School of Public Health, College Station, TX, United States

**Keywords:** Flexible seating, Cardiovascular health, Body mass index, Sedentary behaviour, Standing desk

## Abstract

**Objective:**

Standing desks present a novel approach to reduce sedentary time in the classroom and address cardiovascular risk factors at an early age. In the context of designing a standing desk study, parents and children were surveyed regarding their perceptions and current use of standing desks and other flexible seating.

**Methods:**

Survey administered from January 31st to February 26th, 2024 to a convenience cohort of 50 parent–child pairs presenting for well or acute care at a pediatrics clinic affiliated with an academic institution (Hershey, Pennsylvania, United States). Logistic regression examined parent support of and child willingness to use a standing desk in the classroom.

**Results:**

Parents were primarily non-Hispanic, white females above 40 years of age. Child participants mean age and grade level were 10.5 years and 5th grade respectively. Among parents, 85 % (39/46) were supportive of their child’s use of a standing desk in the classroom, with 4 declining to answer. For children, almost half, 48 % (24/50), were willing to use a standing desk. Acceptability decreased for child body mass index (BMI) ≥85th percentile versus BMI <85th percentile (parent acceptability OR = 0.07 [95 % CI: 0.01–0.63; p = 0.018]; child acceptability: OR = 0.13 [95 % CI 0.03–0.51, p = 0.003]).

**Conclusions:**

Most parents and children are amenable to use of a standing desk in the classroom. Additional information for children with elevated BMI and their parents may be required to address reservations about standing. This study was limited by its small sample size, which may not generalize to other populations.

## Highlights

• Of 50 parent-child pairs, most were willing to support/use classroom standing desks.
• Acceptability decreased by child BMI >85^th^ vs. <85^th^ percentile for both groups.
• Parental support for child standing desk use OR = 0.07 [95 % CI: 0.01–0.63; p = 0.018].
• Child willingness OR = 0.13 [95 % CI 0.03–0.51, p = 0.003]).
• Concerns included discomfort with standing, fidgeting and attention.

## 1 Introduction

Acknowledging a healthy child is a better learner, schools have supported cardiovascular health via physical activity programming (Cornett et al., 2023). These programs have demonstrated moderate success by improving physical activity behavior and fitness (e.g., physical activity minutes, fitness testing outcomes), primarily in elementary grades (Cornett et al., 2023). Yet, it is unclear if changes are maintained once funding ends or staff leaves (Cornett et al., 2023).

Over the past decade, growing evidence suggests that in addition to improved nutrition and greater physical activity, a key component of good health is reduced sedentary time (Wendel et al., 2016, Keadle et al., 2017). Western populations sit an average of 8.5 h/day (Keadle et al., 2017). Children spend most of the school day seated; recess is rare in middle school and almost nonexistent by high school (Wurman, 2024). While physical activity is encouraged via school sports programs, these are not accessible to all students. Classroom modification, specifically standing desks, could impact all students regardless of family socioeconomic status.

Among adults, standing desks demonstrated benefits to cholesterol, triglycerides, blood sugar and blood pressure (Pereira et al., 2020). Wendel et al. demonstrated a beneficial effect on student body mass index (BMI) percentile following two-years of standing desk use among 380 3rd and 4th grade Texas students (Wendel et al., 2016). Benden et al. found students using standing desks had greater caloric expenditure compared to controls, specifically among overweight and obese students (Benden et al., 2011). Additional research on standing desks in schools found academic engagement does not seem to be negatively impacted and may benefit (Dornhecker et al., 2015, Mehta et al., 2015). These experiences and results were noted in studies done worldwide, though high quality randomized trials are needed (Hinckson et al., 2016).

Standing desks fall under the larger umbrella of flexible seating, which includes options such as bean bags, wobble stools and pedal desks. Flexible seating has become increasingly popular in schools. Some educators hope to make the classroom less “institutional”, and others to provide students struggling to sit less distracting ways to move (Cole et al., 2021, Stapp, 2019).

As part of a larger, subsequent standing desk study that will use community-engaged research methods to build partnerships, support the study and enhance results dissemination, we examined parent and child perceptions and current use of flexible seating in classrooms using a survey designed with feedback from the target population.

## 2 Methods

### 2.1 Survey development

Survey questions were designed collaboratively by the research team and pilot tested with five parent–child pairs. Question and response wording was adjusted based on the feedback received. For example, initial response options for screen time included 1–2 hours, 3–4 hours and > 4 hours. These were revised as “up to 2 hours”, “more than 2 hours but less than 4 hours” and “4 or more hours”.

The parent survey consisted of 30 questions on perceptions and experiences with flexible seating, and asked parents to estimate their child’s physical activity and screen time. The child survey consisted of seven questions regarding experiences with and opinions on flexible seating, estimated screen time and sports participation. Children were also asked to rank six different flexible seating options based on their preference: traditional desk, balance ball, standing desk, wobble stool, bean bag chair and pedal desk. Parents and children were referred to Supplemental Fig. 1 in answering the survey. This study was approved by the Penn State College of Medicine Institutional Review Board on January 26th, 2024 (STUDY00023940).

### 2.2 Data collection

Data were collected from January 31st to February 26th, 2024 from a convenience cohort at a pediatric clinic affiliated with an academic medical center (Hershey, Pennsylvania) by two medical students. Surveys were administered on an iPad with direct entry into Research Electronic Data Capture, a secure, online tool to support research studies (Harris et al., 2009). Survey completion constituted participant consent. Parents and children also separately consented and assented respectively to the medical student recording the child’s most recent BMI and BMI percentile from the medical record.

Parent-child pairs were approached either prior to or after being seen by a provider for either well or acute care. Inclusion criteria was as follows: a parent or guardian (18+ years of age) present at the time of the appointment, child between the ages of 5–18 years-old, in kindergarten to 12th grade, attending a brick and mortar (non-remote) school. While “parent” is used throughout the paper for ease, as above, children were eligible to participate if they presented with a parent or other guardian. A goal of 50 completed surveys was set based on available funds.

Medical students read the questions and answers to three parent–child pairs who were not proficient in reading English but wished to participate. In addition, six parents read the questions and answers to their child as they were proficient in reading English, but their child was not. Parent-child pairs were compensated with a $10 gift card.

### 2.3 Statistical analysis

Child willingness to use a standing desk was based on the response to the question, “Would you be willing to use a stand-up desk, even if it was not your first choice?” Response options were yes, no and maybe, which were dichotomized as yes versus no/maybe for the statistical analysis. Parental support of standing desk use was based on the response to the question, “Would you agree to your child using a stand-up desk in the classroom, assuming they were able?” Response options were yes, no and decline to answer. Those who declined to answer were excluded from the analysis.

Logistic regression was used to examine child willingness to use a standing desk by the following variables: BMI, age, race, gender, grade, self-reported screen time, parent flexible seating use and sports participation. Logistic regression was similarly used to examine parental support by the following variables: parent age, education, race, gender, parental use of flexible seating, child BMI and parental report of child’s screen time. For these models, continuous variables were modeled linearly (on the log odds scale) and categorical variables used reference categories. Due to the small sample size, we modeled each variable separately. Odds ratios (ORs) and corresponding 95 % confidence intervals (CIs) and p-values were reported for these models.

## 3 Results

Seventy-two total parent–child pairs were approached to reach the goal of 50 pairs who completed the survey. Reasons for non-participation of these 22 parent–child pairs were not formally recorded, though it was noted those approached at the end of their appointment were more likely to decline. Parents were primarily non-Hispanic, white females, >40 years old and with at least a college degree (Table 1). The mean age of child participants was 10.5 years (range 5–17 years), and mean grade level was 5th grade (range kindergarten to 12th grade).

**Table 1:** Parent and child demographics.

**Demographic variables**	**N (%)**
***Parent***	
**Age**	
≥40 years old	26 (52)
<40 years old	24 (48)

**Gender**	
Male	11 (22)
Female	39 (78)

**Race**	
White	43 (88)
Non-white	6 (12)
Missing	1

**Ethnicity**	
Non-Hispanic	44 (88)
Hispanic	6 (12)

**Education**	
College degree or higher	35 (70)
High school degree or some college	15 (30)

***Child***	
**Gender**	
Male	24 (48)
Female	26 (52)

**Race**	
White	40 (82)
Non-white	9 (18)
Missing	1

**Ethnicity**	
Non-Hispanic	42 (84)
Hispanic	8 (16)

**BMI percentile**	
≥85th percentile	19 (39)
<85th percentile	30 (61)
Missing	1

Most parents, 85 % (39/46), were supportive of their child’s use of a standing desk in the classroom, with 4 declining to answer (Table 2). Parents who were not supportive shared open-ended comments. Representative comments included: “too fidgety when standing”, “worried [child] won’t concentrate and get all work done”, “I am not sure if it will be comfortable”, and “teacher would always be yelling to keep still or no walking around”.

**Table 2:** Parent and child opinions on flexible seating.

**Survey question**	**N (%)**
***Parent***	
**Do you have a job inside or outside of the home that requires you to sit for long periods of time?**	
Yes	35 (71)
No	14 (29)
Missing	1

**Do you currently use some type of flexible seating?**	
Yes	15 (31)
No	33 (69)
Missing	2

**In general, how do you feel about the use of flexible seating to replace traditional classroom seating?**	
Love	28 (56)
Neutral/unsure	19 (38)
Dislike	3 (6)
Missing	0

**Would you agree to your child using a stand-up desk in the classroom, assuming they were able (e.g., no broken legs in a cast or developmental limitations)?**	
Yes	39 (85)
No	7 (15)
Missing	4

**What grades do you think would benefit most from a stand-up desk (check all that apply)?**	
Elementary	24 (62)
Middle	26 (67)
High	29 (74)

**Do you estimate your child currently meets the recommendation for 60 min of physical activity daily during the school week?**	
Yes, definitely	33 (66)
Maybe not every day, but most days	13 (26)
No	4 (8)
Missing	0

**How many hours of daily screen time (TV/video games/social media), not including education material, do you estimate your child gets on the weekdays?**	
Up to 2 h	22 (44)
2 to 4 h	16 (32)
4 or more hours	12 (24)
Missing	0

***Child***	
**Which of the seating options show above (see**Supplemental Fig. 1**) have you used at school in the past (check all that apply)**	
Traditional desk	48 (96)
Balance ball	8 (16)
Standing desk	3 (6)
Wobble stool	11 (22)
Bean bag chair	11 (22)
Pedal desk	1 (2)
Other (open response = table)	1 (2)

**Would you be willing to use a stand-up desk, even if it was not your first choice?**	
Yes	24 (48)
Maybe	15 (30)
No	11 (22)
Missing	0

**How many hours of daily “just for fun” screen time (TV/video games/social media) do you estimate you get on the weekdays?**	
Up to 2 h	17 (35)
More than 2 h but no more than 4 h	19 (40)
4 or more hours	12 (25)
Missing	2

**Do you participate in a sport outside of normal school hours?**	
Yes	39 (78)
No	11 (22)
Missing	0

For children surveyed, almost half, 48 % (24/50), were willing to use a standing desk, with 11 not willing (22 %) and 15 maybe being willing (30 %). The 11 children who indicated they would not use a standing desk had the option to elaborate in an open-ended response. Representative comments included, “don’t like to stand”, “legs hurt standing for whole day”, and “I don’t want to stand”. The bean bag chair was ranked first among flexible seating options by 43 % of children in contrast to the standing desk, which received zero votes as a first choice.

Parent and child factors associated with support for or willingness to use a standing desk respectively were examined using logistic regression (Supplemental Tables 1 and 2). Child BMI percentile (≥85th vs. <85th) was the only variable significantly associated with both parental support for a standing desk and child willingness to use a standing desk. For parents, 65 % (11/17) of those with a child with a BMI ≥85th percentile supported use of a standing desk compared to 96 % (27/28) of those with a child with a BMI <85th percentile, OR = 0.07 (95 % CI: 0.01–0.63, p = 0.018). For children, 21 % (4/19) with a BMI ≥85th percentile and 67 % (20/30) with a BMI <85th percentile indicated willingness to use a standing desk, OR = 0.13 (95 % CI: 0.03–0.51, p = 0.003).

## 4 Discussion

Most parents and children are amenable to the idea of a standing desk in the classroom. Children preferred the bean bag chair as a first choice for flexible seating, but 48 % were willing to use a standing desk. Main objections included concerns about fidgeting and disinterest in standing for a prolonged period. The study findings raise several points for consideration.

Sedentary behavior has been defined as “’a unique set of behaviors with unique environmental determinants and a range of potentially unique health consequences’” (Owen et al., 2010). In pediatric populations, sedentary behavior is associated with adiposity (Barnett et al., 2018). Data remains inconclusive on the dose response between sedentary behaviors and other health outcomes for children, but sedentary habits seem to increase with age (Barnett et al., 2018). Research has demonstrated that pediatric sedentary behaviors track into adolescence and eventually, adulthood (Barnett et al., 2018, Telama et al., 2005).

A 2021 systematic review of flexible seating use compared to a traditional seated desk concluded that flexible seating is a promising intervention to improve health outcomes in children 5–17 years (Guirado et al., 2021). Flexible seating increases overall energy expenditure (e.g., 15–28 % increase for standing desks). Data also suggests improved behavior. For example, children using cycling desks demonstrated increased inhibitory control and improved attention (Guirado et al., 2021). A metanalysis could not be conducted due to the heterogeneous quality of design and results. The authors called for more rigorous studies to inform policy and practice related to flexible seating use for children and adolescents (Guirado et al., 2021).

Our survey was specifically concerned with the acceptability of standing desks for students, and it was notable that for children with a BMI ≥85th percentile both parent and child were significantly less likely to be amenable to a standing desk. However, initial work suggests that children with an elevated BMI also may have more to gain from standing desk use. Among students enrolled in a standing desk intervention, caloric expenditure was 17 % greater for those in the treatment arm, but the subset of participants with a BMI above the 85th percentile had a 32 % increase in caloric expenditure compared to controls (Benden et al., 2011). Reluctance to use a standing desk may be overcome by changing the “normal”. A qualitative study of parents and children regarding sedentary behavior identified some of the impetus for sitting as the “normal” or “habit” based on the existing environment (Hidding et al., 2017). This may explain some of the appeal of the bean bag chair; while a flexible option in the classroom, it supports a sedentary position. Parents and children should be told to anticipate children may feel tired and have minor complaints as they acclimate to standing. While multiple standing desk studies report no difference or improved pain compared to controls, in one study with secondary students, 51 % reported back and leg pain (Guirado et al., 2021, Sudholz et al., 2016).

Study limitations include a small sample size, which may have limited the ability to detect differences in willingness to use a standing desk by age, and potential selection bias, as those who completed the survey may be different than those who were not interested. Data are from one pediatric clinic and may not be generalizable to other settings. Additionally, parents and children were predominantly non-Hispanic white, thus the perspectives of other groups may not be well represented. While the survey questions were modified with parent and child feedback, results would have been strengthened by the use of validated instruments, specifically to assess physical activity and screen time. Finally, as noted above, there were some cases where parents helped their children read questions, which may also have introduced bias to their responses.

## 5 Conclusion

In summary, these data provide a snapshot of parent and child perspectives on flexible seating, particularly standing desks. Parents and children were overall amenable to standing desk use, with decreased support and willingness with child BMI ≥85th percentile. This information will be valuable in designing a standing desk intervention, especially in considering how information should be presented for children with elevated BMI and their parents who may have greater reservations about standing. Further, the association of race, sedentary time and other demographics with willingness to use standing desks should be explored in a larger study.

## 6 Human participant compliance statement

Approval was obtained by the Penn State College of Medicine Institutional Review Board.

## 7 Data sharing

Data requests may be made by direct communication with the corresponding author.

## CRediT authorship contribution statement

**Katie L. Wasserstein:** Writing – review & editing, Writing – original draft, Project administration, Methodology, Investigation, Formal analysis. **Meghan L. Shah-Hartman:** Writing – review & editing, Visualization, Project administration, Methodology, Investigation, Formal analysis. **W. Gavin Luzier:** Writing – review & editing, Visualization, Validation, Formal analysis, Data curation. **Eric W. Schaefer:** Writing – review & editing, Visualization, Validation, Formal analysis, Data curation. **Mark E. Benden:** . **Deepa L. Sekhar:** Writing – review & editing, Validation, Supervision, Project administration, Methodology, Funding acquisition, Formal analysis, Conceptualization.

## Declaration of competing interest

The authors declare that they have no known competing financial interests or personal relationships that could have appeared to influence the work reported in this paper.

## Data Availability

Data will be made available on request.
