# Supplementary material for: Parent and child opinion on the use of standing desks in the classroom
Source: Prev Med Rep. 2024 Aug 30;46:102875. doi: 10.1016/j.pmedr.2024.102875 (PMC11404221; doi:10.1016/j.pmedr.2024.102875)
Supplement: Supplementary Data 1 [file mmc1.docx]

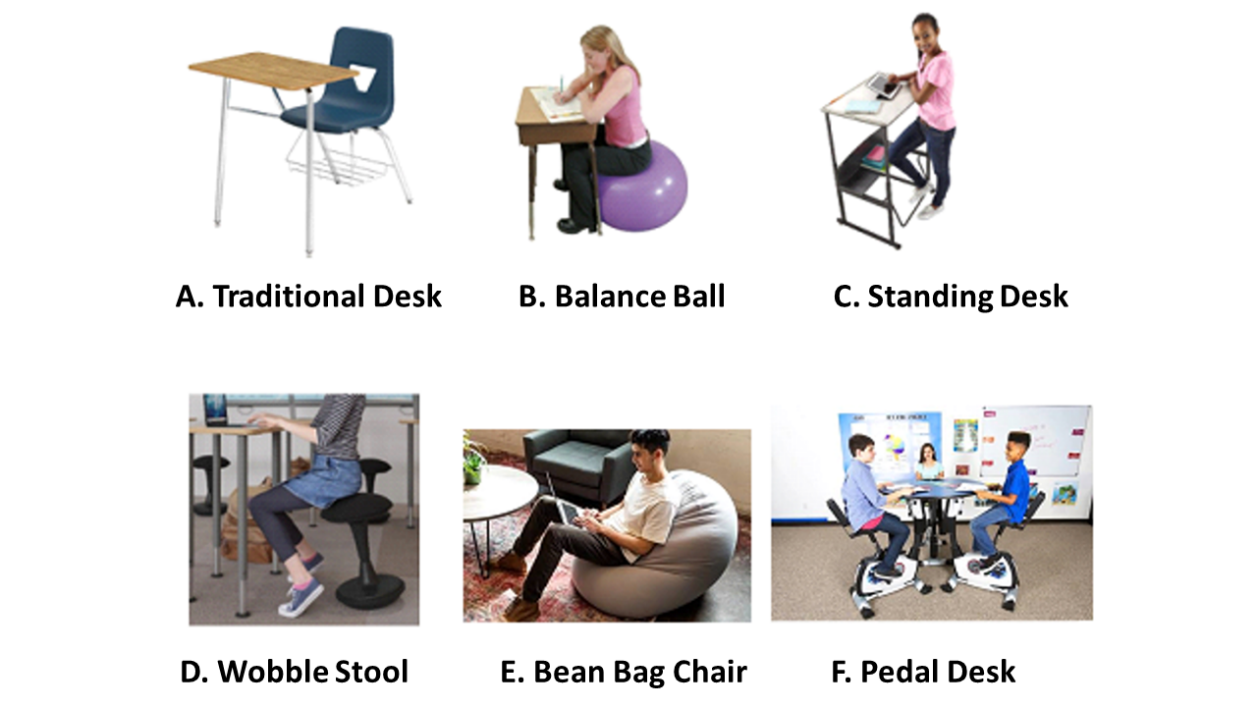


**Supplemental Figure 1.** Traditional and flexible seating graphics presented in the parent-child survey^1-6^

Figure image sources:

1. Student Combination Desk. Wayfair. <https://www.wayfair.com/Lorell--Combination-Desk-99914-L271-K~LRO10150.html?refid=GX418622807237-LRO10150&device=c&ptid=841525430854&network=g&targetid=pla-841525430854&channel=GooglePLA&ireid=99404096&fdid=1817&gclid=Cj0KCQjw4bipBhCyARIsAFsieCxvGewhlwFtqtjt6ZndE347l64EXjjhNuL1cXrbr0oiD3WLi2u64RIaAo2PEALw_wcB>. Accessed April 15, 2024.
2. Abiliations StayN’Place Ball, 37 Inches, Color May Vary. School Specialty. <https://www.schoolspecialty.com/abilitations-staynplace-ball-37-inches-color-may-vary-1318000?utm_source=google&utm_medium=shopping&utm_campaign=18275001835&product_id=1318000&ad_group_id=139408759325&feed_item_id=&target_id=pla-63187294797&gclid=Cj0KCQjw4bipBhCyARIsAFsieCxGOORts87ZrRZbmtXnp3INXhD-WLtRx1Y0SikIzkJn77I7OOZe4gIaArknEALw_wcB&keyword=&kxconfid=u7avswvjn&source=ggl&campaignid=WP_-_PPC_-_GGL_-_Shopping_-_AP_-_ALL_-_NT_-_PMRD_-_RVN_-_G_-_US&placement=shopping&keyword>. Accessed April 15, 2024
3. Safco Alphabetter Adj Stand-Up Desk 36 x24 x42 Black 1206BE. https://www.frysfood.com/p/safco-alphabetter-adj-stand-up-desk-36-x24-x42-black-1206be/0007355512064. Accessed April 15, 2024.
4. Delacora Adjustable Height 13’’ Wide Active Padded Sit Stand Office Classroom Wobble Stool with Rocking and Tilting Motion. <https://www.lightingdirect.com/delacora-ff-ay8001-office-wobble-stool-adjustable-height-13-wide-active-padded-sit-stand-office-classroom-wobble-stool-with-rocking/p4276070>. Accessed April 15, 2024
5. Entrepreneur Deals, *Moon Pod.* Entrepreneur. <https://www.entrepreneur.com/business-news/this-re-engineered-bean-bag-is-all-the-rage/341962>. Published November 8, 2019. Accessed April 17, 2024.
6. KIDSFIT KC-758 Three Person Round Pedal Desk With Resistance. School Specialty. <https://www.schoolspecialty.com/kidsfit-kc-758-three-person-round-pedal-desk-with-resistance-1577615>. Accessed April 15, 2024.
