# Supplementary material for: Parent and child opinion on the use of standing desks in the classroom
Source: Prev Med Rep. 2024 Aug 30;46:102875. doi: 10.1016/j.pmedr.2024.102875 (PMC11404221; doi:10.1016/j.pmedr.2024.102875)
Supplement: Supplementary Data 2 [file mmc2.docx]

**Supplemental Table 1.** **Logistic regression model of parent willingness to support flexible seating use**

| **Variable** | **OR** | **95% CI** | **p-value** |
| --- | --- | --- | --- |
| Parent age >40 years  <40 years (ref) | 1.56 | 0.31-7.89 | 0.59 |
| Father  Mother (ref) | 2.07 | 0.22-19.3 | 0.52 |
| Parent race white  Parent race non-white (ref) | 7.20 | 0.82-63.2 | 0.08 |
| College degree or higher  High school or some college (ref) | 2.17 | 0.41-11.4 | 0.36 |
| Parent uses flexible seating  Does not use flexible seating (ref) | 0.89 | 0.14-5.53 | 0.90 |
| Child BMI >85^th^%  Child BMI <85^th^% (ref) | **0.07** | **0.01-0.63** | **0.018** |
| Parent report of child screen time of >4 hours  >2 to <4 hours  <2 hours (ref) | 0.79  1.15 | 0.11-5.66  0.17-7.90 | 0.82  0.90 |
