# Supplementary material for: Parent and child opinion on the use of standing desks in the classroom
Source: Prev Med Rep. 2024 Aug 30;46:102875. doi: 10.1016/j.pmedr.2024.102875 (PMC11404221; doi:10.1016/j.pmedr.2024.102875)
Supplement: Supplementary Data 3 [file mmc3.docx]

**Supplemental Table 2. Logistic regression model of child willingness to use flexible seating**

| **Variable** | **OR** | **95% CI** | **p-value** |
| --- | --- | --- | --- |
| Age (by 1-year age increase) | 0.99 | 0.85-1.16 | 0.94 |
| Male  Female (ref) | 1.17 | 0.38-3.54 | 0.79 |
| White  Non-white (ref) | 3.87 | 0.71-20.9 | 0.12 |
| Grade (by one grade level increase) | 0.99 | 0.85-1.15 | 0.91 |
| BMI >85^th^%  BMI <85^th^% (ref) | **0.13** | **0.03-0.51** | **0.003** |
| Child-reported screen time of >4 hours  >2 to <4 hours  <2 hours (ref) | 0.27  0.49 | 0.06-1.30  0.13-1.88 | 0.10  0.30 |
| Parent uses flexible seating  Does not use flexible seating (ref) | 1.21 | 0.36-4.12 | 0.76 |
| Participation in sports  No sports participation (ref) | 1.84 | 0.46-7.32 | 0.39 |
